# Supplementary material for: Radtools: R utilities for convenient extraction of medical image metadata
Source: F1000Res. 2019 Mar 25;7:ISCB Comm J-1976. Originally published 2018 Dec 24. [Version 3] doi: 10.12688/f1000research.17139.3 (PMC6518432; doi:10.12688/f1000research.17139.3)
Supplement: Peer review history

# Peer review: Reviewer response for version 3

**DOI:** 10.5256/f1000research.20262.r46122

**Published:** 2019-05-14

**Authors:** Volker Schmid

**Affiliations:** 1 Department of Statistics, Ludwig Maximilian University of Munich, Munich, Germany

My concerns have been addressed appropriately. The vignettes now nicely show the value of the Radtools package. Thanks for all the effort you put into the package and the paper.

I have read this submission. I believe that I have an appropriate level of expertise to confirm that it is of an acceptable scientific standard.

# Peer review: Reviewer response for version 3

**DOI:** 10.5256/f1000research.20262.r46121

**Published:** 2019-03-26

**Authors:** Andrey Fedorov

**Affiliations:** 1 Department of Radiology, Brigham and Women's Hospital, Harvard Medical School, Boston, MA, USA

The revised manuscript now includes a demonstration and specific summary of how the newly introduced package is different from the already existing functionality. Thank you for this revision and your persistence! I believe this improved the quality of the article sufficiently to make it suitable for indexing.

I have read this submission. I believe that I have an appropriate level of expertise to confirm that it is of an acceptable scientific standard.

# Peer review: Reviewer response for version 2

**DOI:** 10.5256/f1000research.19528.r43610

**Published:** 2019-02-15

**Authors:** Volker Schmid

**Affiliations:** 1 Department of Statistics, Ludwig Maximilian University of Munich, Munich, Germany

Unfortunately the authors have revised their manuscript only slightly. I still see a general interest in the aim and the idea of the provided package. However, the value of the package is not explained in the manuscript, and that may be because the ability of the package is (still) limited. The manuscript needs example code, Figures, Tables etc. to show the value of the package.

 The manuscript needs to answer the following question: Why is it convenient to use your package compared to writing code specifically tailored for my data set?   

I have read this submission. I believe that I have an appropriate level of expertise to state that I do not consider it to be of an acceptable scientific standard, for reasons outlined above.

# Peer review: Reviewer response for version 2

**DOI:** 10.5256/f1000research.19528.r43611

**Published:** 2019-02-12

**Authors:** Andrey Fedorov

**Affiliations:** 1 Department of Radiology, Brigham and Women's Hospital, Harvard Medical School, Boston, MA, USA

Unfortunately, the manuscript is still lacking details about the added value it provides as compared to oro.* packages. The clarification that it provides "convenient extraction and exploration of image metadata", which, according to authors, "is not provided by existing tools", is not helpful. I do not understand what that statement actually means. Please include specific details how the functionality provided by Radtools is different from oro.*, and justify why that new functionality is important. 

 The authors refer to the "tests" directory in the package source, but the datasets referenced are from a local Dropbox folder. Considering TCIA provides API for retrieving images, it would make more sense to include code that retrieves all of the tests. Also, it is not clear why the specific datasets were selected, how they are different, and what aspects of the implementation they are testing.

 About the updated title: "smooth" is equivalent to "convenient". Both are subjective qualifiers. I understand it was the authors intent to make extraction and navigation "more smooth" than available alternatives in their judgement, but whether this was successful or not will be up to the users of the package. I would drop the subjective qualifier, unless the manuscript includes specific objective criteria that would demonstrate it is "more smooth" or "more convenient" than the alternatives. Not sure how that would be demonstrated though - perhaps blinded user surveys?

I have read this submission. I believe that I have an appropriate level of expertise to state that I do not consider it to be of an acceptable scientific standard, for reasons outlined above.

# Peer review: Reviewer response for version 1

**DOI:** 10.5256/f1000research.18738.r42220

**Published:** 2019-01-07

**Authors:** Volker Schmid

**Affiliations:** 1 Department of Statistics, Ludwig Maximilian University of Munich, Munich, Germany

This manuscript describes the R package radtools. The aim of the R package is "smooth navigation of medical image data".

 The idea to provide functions which appear "smooth" for the end user is of great importance. However, the functions provides in the package do not seem to be of much (additional) benefit to the end user. Functions for reading NIfTI and DICOM images are wrappers around function in the packages oro.nifti and oro.dicom; visualisations of medical images are realised in oro.nifti. Only the functions for exploring DICOM headers are genuinely original. This is of course an important part of working with (DICOM) images.

 From my understanding, F1000Research requires software tools articles to contain examples of the use of the tools. This manuscript does not contain any examples. An example (or two examples) would not only strengthen the manuscript, but could/would also show the benefit of the R package itself.

I have read this submission. I believe that I have an appropriate level of expertise to state that I do not consider it to be of an acceptable scientific standard, for reasons outlined above.

# Peer review: Reviewer response for version 1

**DOI:** 10.5256/f1000research.18738.r42221

**Published:** 2019-01-07

**Authors:** Andrey Fedorov

**Affiliations:** 1 Department of Radiology, Brigham and Women's Hospital, Harvard Medical School, Boston, MA, USA

The authors present a new R package developed to support the use of DICOM and NIfTI files from the R environment. The authors rightfully discuss the popularity of R and the need to support image processing tasks in this environment. The argument for development of the proposed package, radtools, is that "[...] no existing package makes image metadata conveniently accessible. Extracting image metadata, combining across slices, and converting to useful formats can be prohibitively cumbersome, especially for DICOM". The resulting package is available from CRAN, and this reviewer confirmed its installation and basic functions.

 The major issues that need to be addressed to make the article sound are the following:

1. Justification of the development of a new package for working with DICOM, or with NIfTI, is not sufficient.
2. No details are provided about how the functionality was tested, and about the capabilities and limitations of the package in terms of supporting specific DICOM objects.
3. Related to 2), no details are provided about how the DICOM files are handled "under the hood", i.e., whether all IO functionality was implemented from scratch, or the package is using some other DICOM libraries.

Through the text, the authors reference other R packages for similar tasks, and most notably oro.dicom and oro.nifti ^1^. Those packages have been around for quite a long time, are broadly used, based on citations of the corresponding articles, and arguably provide the functionality of the proposed new package (loading data in the aforementioned formats, examination of the attributes, visualization of the images), plus more (e.g., writing of the NIfTI data).

 DICOM is a complex standard, with a lot of ways information can be stored. For example, there are different methods to encode the content (transfer syntax), different character sets that can be used, private attributes. Therefore, often the quality of a DICOM implementation is defined to a large degree by the data that was used to test the implementation. The quality is also usually improved over time with the usage of the implementation. The proposed package is not accompanied by any details about what types of DICOM objects are supported, what was tested and how. Given it is a new package with a short development and usage history, one has to make a very strong argument for introducing such new tools in presence of existing alternatives.

 Other suggestions:

- The discussion of the DICOM objects is an oversimplification, which is reflected in the implementation of the functionality. The standard defines various types of objects that can be serialized as files, but those objects are not limited to images. As an example, DICOM defines Structured Reporting object, which will not have PixelData. The proposed package fails to read such object. The authors can find sample SR objects in the familiar to them TCIA (e.g., see QIN-HEADNECK collection).
- "smooth" is a subjective qualifier that is redundant in the title and the text.

I will be happy to reconsider this article after the authors address the above concerns. But my current opinion is that oro.dicom and oro.nifti set a rather high bar for any new implementation of similar functionality.

I have read this submission. I believe that I have an appropriate level of expertise to state that I do not consider it to be of an acceptable scientific standard, for reasons outlined above.

## References

[1] WhitcherBSchmidVThorntonA: Working with the DICOM and NIfTI Data Standards inR. *Journal of Statistical Software*.2011;44(6) : 10.18637/jss.v044.i06 10.18637/jss.v044.i06
